# Optical Microfibre Based Photonic Components and Their Applications in Label-Free Biosensing

**DOI:** 10.3390/bios5030471

**Published:** 2015-07-22

**Authors:** Pengfei Wang, Lin Bo, Yuliya Semenova, Gerald Farrell, Gilberto Brambilla

**Affiliations:** 1 Photonics Research Centre, Dublin Institute of Technology, Kevin Street Dublin 8, Dublin, Ireland; E-Mails: bo.lin@mydit.ie (L.B.); yuliya.semenova@dit.ie (Y.S.); gerald.farrell@dit.ie (G.F.); 2 Optoelectronics Research Centre, University of Southampton, Southampton SO17 1BJ, UK; E-Mail: gb2@orc.soton.ac.uk

**Keywords:** fibre optics, optical microfibre, label-free biosensing

## Abstract

Optical microfibre photonic components offer a variety of enabling properties, including large evanescent fields, flexibility, configurability, high confinement, robustness and compactness. These unique features have been exploited in a range of applications such as telecommunication, sensing, optical manipulation and high Q resonators. Optical microfibre biosensors, as a class of fibre optic biosensors which rely on small geometries to expose the evanescent field to interact with samples, have been widely investigated. Due to their unique properties, such as fast response, functionalization, strong confinement, configurability, flexibility, compact size, low cost, robustness, ease of miniaturization, large evanescent field and label-free operation, optical microfibres based biosensors seem a promising alternative to traditional immunological methods for biomolecule measurements. Unlabeled DNA and protein targets can be detected by monitoring the changes of various optical transduction mechanisms, such as refractive index, absorption and surface plasmon resonance, since a target molecule is capable of binding to an immobilized optical microfibre. In this review, we critically summarize accomplishments of past optical microfibre label-free biosensors, identify areas for future research and provide a detailed account of the studies conducted to date for biomolecules detection using optical microfibres.

## 1. Introduction

Generally, a biosensor is an integrated device which is designed to detect or quantify bacteria, viruses or a variety of biochemical molecules including specific DNA sequences, proteins, enzymes, antibodies and oligonucleotides. Many biosensors developed to date are affinity-based, meaning that an immobilized capture probe is adopted to bind the molecule being sensed—the target or analyte—selectively, therefore transferring the challenge of detecting a target in solution into detecting a change at a localized surface. This change can then be measured in a variety of ways, such as changes in evanescent field and surface plasmon resonance spectra. So far, biosensors have found many applications, including biomarker detection for medical diagnostics [1], pathogen [2] and toxin detection in food and water [3].

Given the well-known advantages of optical fibre based sensors, such as compact size, high resolution, immunity to electromagnetic interference and the potential for remote operation, several optical fibre based biosensors have been developed. However, the majority of the proposed fibre optic biosensors require the use of a label which is a molecule (e.g., a fluorophore) chemically attached to aid the detection of a target molecule [4]. The process of labelling can significantly increase complexity thus label-free detection is preferable in many bio-related applications. For this reason, research has focused in recent years on the label-free detection technique, which refers to that special typology of biosensing wherein the detection of the chemical agent or biological (interaction/agent) by the transducer does not rely on dyes, enzymes or radiolabels. Label-free fibre optic biosensors use optical fibres as the transduction element, and rely exclusively on optical transduction mechanisms for detecting target biomolecules [5]. One reliable and sensitive optical method is evanescent field sensing. Several label-free biosensors have been recently demonstrated which consist of simple structures based on non-adiabatic tapered optical fibres. Such sensors utilize the evanescent field of the optical microfibre uniform waist region to enhance the light-biomaterial interaction for biosensing applications.

**Figure 1:**
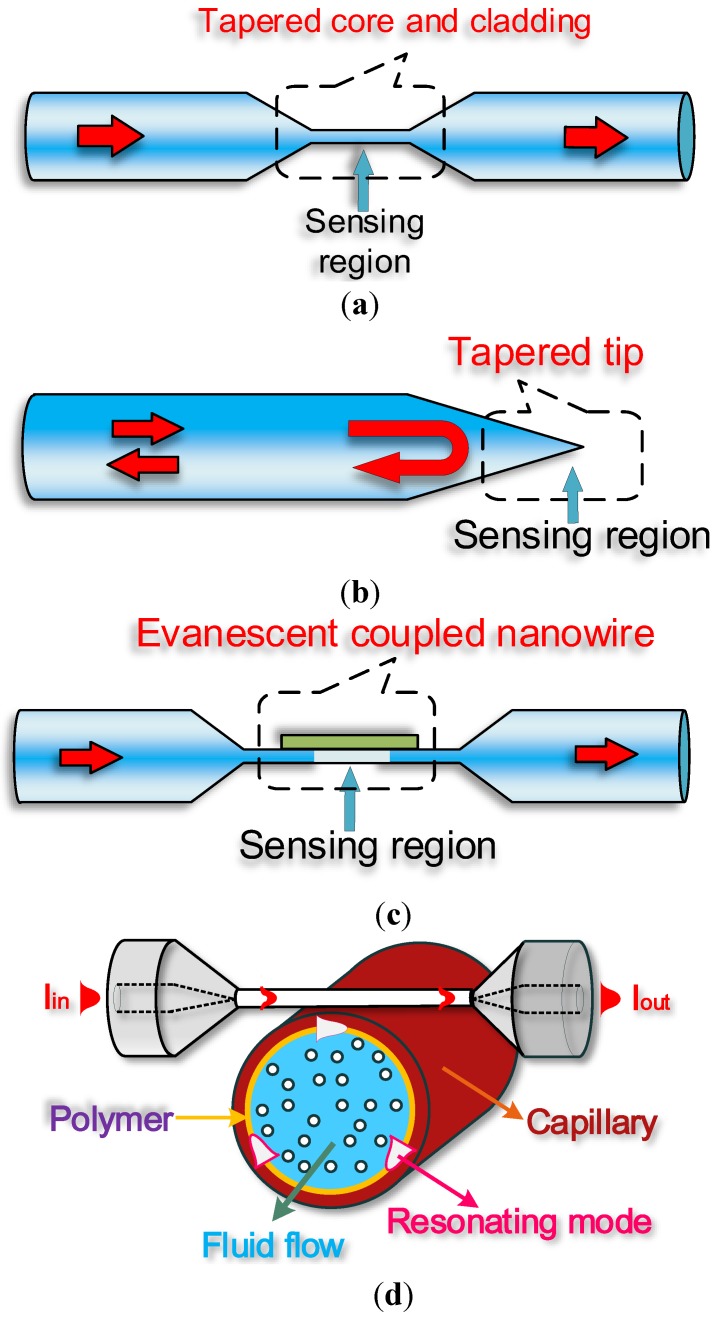
Illustration of typical structures of optical microfibre sensors: (**a**) straight microfibre biosensor; (**b**) microfibre tip biosensor; (**c**) evanescently coupled microfibre sensor; (**d**) heterogeneous resonant sensor.

Optical microfibres, also known as optical/photonic microwires, are optical fibre tapers with a uniform waist region size comparable to the wavelength of light propagating in them. Optical microfibres are usually fabricated by heating [6] regular-sized optical fibres under a small degree of tension. The result is a biconical taper that provides a smooth lossless connection to other fiberized components. By controlling the pulling rate during the fabrication process, the taper profile can be accurately controlled to suit specific applications [7]. Optical materials other than silica have been used to fabricate optical microfibres, including phosphate [8], tellurite [9], lead silicate [10], bismuthate [11] and chalcogenide glasses [12] and a variety of polymers [13,14,15]. The remarkable optical and mechanical properties exhibited by optical microfibres are constantly exploited for optical sensing and include large evanescent fields, strong optical confinement, flexibility, configurability and robustness. Such desirable characteristics have gathered much attention in recent years and made optical microfibres an excellent platform for developing label-free optical biosensors. In the next sections, a summary of optical microfibre fabrication and properties will be presented, followed by an analysis of different typologies of label-free biosensors. In particular, label-free biosensors based on optical microfibres will be categorized in four groups according to the configurability they exploit: straight tapered microfibre biosensors, microfibre tip biosensors, evanescently coupled microfibre sensors and heterogeneous resonant sensors. The schematics of these optical microfibre geometries are shown below in Figure 1:

Figure 1a schematically represents a tapered optical microfibre which tightly confines an optical beam to a small transverse area and provides for a strong interaction with the surrounding medium which can be used for a biosensing detection. This basic photonic component has opened up new opportunities for a wide range of applications including high-sensitivity optical sensors, nonlinear optics, atom trapping, micro/nano-scale photonic devices and applications involving evanescent coupling to planar waveguides or microcavities *etc.*; Figure 1b indicates a microfibre tip structure that can be developed into a microfibre biosensing probe due to the strong light confinement at the end of the tip. The interaction between the confined light and the surrounding medium can be determined by measuring the changes in the back reflection of this structure. The photonic component offers excellent potential for sensing inside subwavelength liquid droplets, bubbles or biocells because the unique probe structure possesses a three dimensional detection capability and a compact size; Figure 1c shows an optical microfibre coupled with the other optical microfibre forming the well-known optical microfibre coupler. Mode coupling between the lower-order symmetric and anti-symmetric supermodes strongly occurs between the two coupling microfibre arms, and therefore this structure can underpin potential applications in the field of high performance fibre sensors; Figure 1d presents the schematic of an optical microfibre heterogeneous resonant sensor in which an optical microfibre acts as a coupling waveguide to excite or collect light from a range of high-Q whispering gallery mode microresonators such as micro-disks, micro-rings, micro-spheres, micro-toroids, micro-capillaries or micro-bottle resonators. By matching the propagation constants of the modes propagating in the optical microfibre and the microresonator, coupling efficiencies in excess of 90% have been achieved previously [16]. Evanescent sensing in these types of high-Q microresonators has been widely used for detecting biological targets positioned in proximity of the microresonator surface.

## 2. Waveguiding Properties of Optical Microfibres

Normally, optical fibre consists of a cylindrical core and a surrounding cladding, both made of silicate glasses. According to the waveguiding theory, light propagating through an optical fibre consists of two components: the guided field in the core and the exponentially decaying evanescent field in the cladding. In a conventional uniform-diameter telecom optical fibre, the evanescent field decays to almost zero within the cladding. Therefore, light propagating in uniform-diameter cladded fibres cannot interact with the surrounding medium. For example, a singlemode optical fibre (SMF) is often approximated as a step-index core-cladding structure. The typical diameters of the core and cladding of a telecom SMF (such as SMF28 produced by Corning) are 9 μm and 125 μm, respectively. The refractive index of the fibre core is slightly higher than that of the cladding to achieve a weakly-guiding condition [17]. Due to the small refractive index difference between the core and cladding, the weakly-guided fundamental modes have a mode field diameter that exceeds that of the core and thus extends into the cladding with the result that a substantial fraction of the light propagates within the cladding as evanescent field. However, this evanescent field is not accessible for sensing purposes due to the relatively large diameter of the cladding relative to the mode field diameter. In the case of optical microfibres, the total fibre diameter is reduced to a few micrometres and the telecom fibre silica cladding now acts as guiding core, with the surrounding material acting as cladding. The evanescent field can extend into the surrounding medium, effectively propagating outside the fibre. It is by this means that the evanescent field of an optical microfibre is accessible and can be utilized for sensing.

It should be noted that the diameter of an optical microfibre is greatly reduced so the original fibre core at the waist region is of negligible dimension. Moreover, during tapering core dopants diffuse across the whole microfibre cross section, providing a uniform dopant concentration. Therefore, an optical microfibre can be considered as a cylindrical waveguide with a homogeneous RI profile. The surrounding environment becomes the microfibre cladding. Since a significant proportion of light propagates outside of the microfibre, it is necessary for the cladding media to have low RIs to achieve an adequate level of confinement for light propagating along the microfibre. Depending on the proposed application, typical “claddings” for microfibres can be air, water or low-index polymer materials.

The propagating electric and magnetic fields of an optical microfibre can be calculated by solving Maxwell’s equations in a similar manner to that used for a standard SMF [18]. However, due to the high contrast of the refractive indices between the optical microfibre and the environment-cladding, the weakly-guiding approximation (where the index difference between the core and cladding is close to 1%) used for standard SMF cannot be generally used. Therefore, light propagation along an optical microfibre needs to be calculated by a rigorous vectorial solution of Maxwell’s equations for a uniform cylindrical waveguide with a homogeneous index profile.

Due to the large index difference between the core and cladding (30% for silica in air), optical microfibres with relatively large diameters (a few micrometers) can confine and support the transmission of several higher order modes. At a fixed wavelength, only microfibres with diameters that fall below certain values (normally smaller than the wavelength) can support single-mode guidance. Fortuitously, this dimension criterion for single-mode guidance does not hold strictly for adiabatically tapered optical microfibres, where the coupling between the fundamental mode and higher order modes is negligible throughout the length of the tapered microfibre. Adiabatically tapered optical fibres even with relatively large diameters, due to the slow variation of diameter along the transition region, can support the propagation of the sole launched fundamental mode along the tapers. Therefore, only the electric field distribution of the fundamental HE_11_ mode is considered for mode field analysis, since all the tapered microfibres discussed in this review are adiabatic in nature.

Since the refractive index difference between the two media acting as core and cladding of the microfibre is substantial, the exact solutions of Maxwell’s equation for the transverse electric (TE_0m_), the transverse magnetic (TM_0m_), and the hybrid modes (HE_vm_ or EH_vm_) give rise to the following eigenvalue equations, respectively [19]:

(1) $$\left[\frac{J_{1}\left(U\right)}{UJ_{0}\left(U\right)}+\frac{K_{1}\left(W\right)}{WK_{0}\left(W\right)}\right]=0$$

(2) $$\left[\frac{n_{1}^{2}J_{1}\left(U\right)}{UJ_{0}\left(U\right)}+\frac{n_{2}^{2}K_{1}\left(W\right)}{WK_{0}\left(W\right)}\right]=0$$

and

(3) $$\left[\frac{J_{1}^{'}\left(U\right)}{UJ_{1}\left(U\right)}+\frac{K_{1}^{'}\left(W\right)}{WK_{1}\left(W\right)}\right]\left[\frac{J_{1}^{'}\left(U\right)}{UJ_{1}\left(U\right)}+\frac{n_{1}^{2}K_{1}^{'}\left(W\right)}{n_{2}^{2}WK_{1}\left(W\right)}\right]={\left(\frac{\nu \beta }{kn_{1}}\right)}^{2}{\left(\frac{V}{UW}\right)}^{4}$$

where *J*_ν_ is the *v*-th Bessel function of the first kind and *K*_ν_ is the *v*-th modified Bessel function of the second kind, $U=a\sqrt{k^{2}n_{1}^{2}-\beta^{2}}$, $W=a\sqrt{\beta^{2}-k^{2}n_{2}^{2}}$, *a* is the microfibre radius, *k* = 2π/λ, *n_1_* and *n_2_* are the RIs of the microfibre and the surrounding medium, respectively, *β* is the propagation constant and $V=\sqrt{U^{2}+W^{2}}\;$ the normalized frequency.

Using a cylindrical coordinate system (*r*, θ, *z*), the electric field distributions for the HE_11_ modes are expressed as [19]:

(4) $$E_{r}=\begin{array}{cc} \{\begin{array}{c} -\frac{a_{1}J_{0}\left(U\frac{r}{a}\right)+a_{2}J_{2}\left(U\frac{r}{a}\right)}{J_{1}\left(U\right)}\cdot f_{1}(\theta )\left(0<r\leq a\right)\\ -\frac{U}{W}\frac{aK_{0}\left(W\frac{r}{a}\right)-a_{2}K_{2}\left(W\frac{a}{r}\right)}{K_{1}\left(W\right)}\cdot f_{1}\left(\theta \right)\left(a<r<\infty \right) \end{array} & \end{array}$$

(5) $$E_{\theta }=\begin{array}{cc} \{\begin{array}{c} -\frac{a_{1}J_{0}\left(U\frac{r}{a}\right)-a_{2}J_{2}\left(U\frac{r}{a}\right)}{J_{1}\left(U\right)}\cdot g_{1}(\theta )\left(0<r\leq a\right)\\ -\frac{U}{W}\frac{aK_{0}\left(W\frac{r}{a}\right)+a_{2}K_{2}\left(W\frac{a}{r}\right)}{K_{1}\left(W\right)}\cdot g_{1}\left(\theta \right)\left(a<r<\infty \right) \end{array} & \end{array}$$

(6) $$E_{z}=\begin{array}{cc} \{\begin{array}{c} -\frac{iU}{a\beta }\frac{J_{1}\left(U\frac{r}{a}\right)}{J_{1}\left(U\right)}\cdot f_{1}(\theta )\left(0<r\leq a\right)\\ -\frac{iU}{a\beta }\frac{K_{1}\left(W\frac{r}{a}\right)}{K_{1}\left(W\right)}\cdot f_{1}(\theta )\left(a<r<\infty \right) \end{array} & \end{array}$$

where *a* is the optical microfibre radius, and

$$a_{1}=\frac{V}{2UW\left(b_{1}+b_{2}\right)}-\frac{1}{2}，\;\;a_{2}=\frac{V}{2UW\left(b_{1}+b_{2}\right)}+\frac{1}{2}$$

$$b_{1}=\frac{1}{2U}\left[\frac{J_{0}\left(U\right)}{J_{1}\left(U\right)}-\frac{J_{2}\left(U\right)}{J_{1}\left(U\right)}\right]，\;\;b_{2}=\frac{1}{2U}\left[\frac{K_{0}\left(W\right)}{K_{1}\left(W\right)}-\frac{K_{2}\left(W\right)}{K_{1}\left(W\right)}\right]$$

$$f_{1}\left(\theta \right)=\sin\left(\theta \right)，\;\;g_{1}\left(\theta \right)=\cos\left(\theta \right)$$

**Figure 2:**
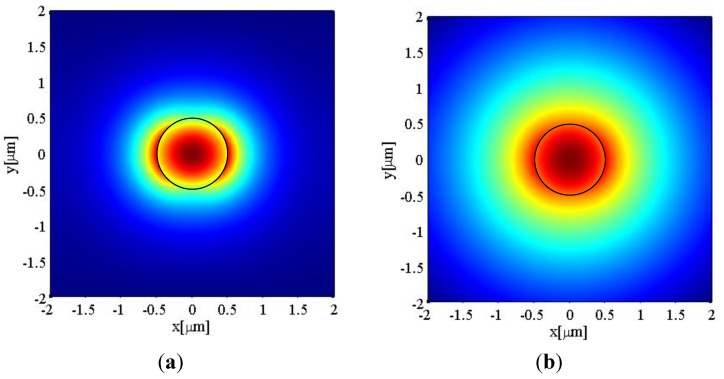
Electric field distribution for a 1 μm diameter silica microfibre when the refractive index of the surrounding medium is (**a**) *RI* = 1; and (**b**) *RI* = 1.33. The black circle indicates the diameter of the microfibre.

As an example of the use of the analysis above, the electric field components of the HE_11_ mode at the wavelength λ = 1550 nm are calculated using a commercial software—Comsol. The electric field intensity *E^2^*, defined as *E_r_^2^* + *E*_θ_*^2^* + *E_z_^2^*, of a 1 µm diameter microfibre with an air cladding (*RI* ≈ 1) is shown below in Figure 2a. One can see that a portion of the light is guided as the evanescent field outside of the microfibre with a power discontinuity at the fibre surface. This evanescent field fades away within a distance of circa 1 μm from the microfibre surface. A second example important for many sensing applications concerns the case when microfibres operate in an aqueous environment. The electric field intensity for the same microfibre with water cladding (*RI* = 1.33) is shown in Figure 2b. In comparison with the air-clad microfibre, the smaller index difference between water and silica results in a weaker confinement of the guided modes in the silica microfibre. Also Figure 2b shows that the electric field expands further outside the microfibre with a longer tail.

The ratio of the power fraction in the microfibre to the total guided power is denoted as η and calculated as:

(7) $$\eta =\frac{\int_{0}^{a}S_{z}dA}{\int_{0}^{a}S_{z}dA+\int_{a}^{\infty }S_{z}dA}$$

where *S_z_* is the longitudinal component of Poynting vector and dA=r⋅dr⋅dθ.

**Figure 3:**
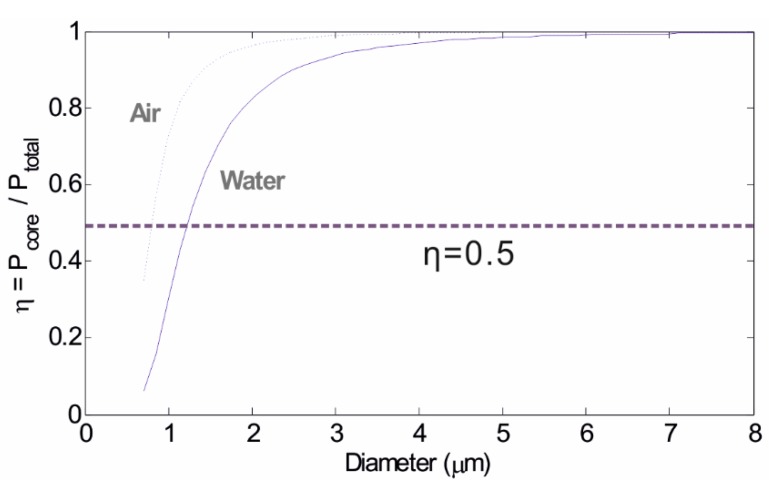
Change of the fraction of the guided power in the microfibres with different microfibre diameters. The dashed line and the solid line correspond to microfibres with air cladding and water cladding, respectively.

Figure 3 shows the change of η with the microfibre diameter for both air-clad and water-clad conditions. A low value of η corresponds to a large evanescent field. For microfibres with air cladding, η starts to decrease when the microfibre diameter falls below circa 4.5 µm and, as the diameter keeps reducing, η decreases more sharply. The power fraction of the evanescent field exceeds the power within the microfibre when the diameter drops to approximately 0.8 µm. In comparison, microfibres with water cladding show smaller values of η because the smaller index contrast between water and silica results in a weaker mode confinement. η reaches 0.5, the point where the power in the evanescent field becomes larger than that in the core, at a diameter of approximately 1.25 µm for silica in water. In other words, microfibres with water cladding show larger evanescent field fractions than those with air cladding due to the low RI contrast between the waveguide and the surrounding medium [20].

In general, the fraction of the evanescent field increases with decreasing of microfibre diameters. Therefore, microfibres with smaller diameters can facilitate stronger light-environment interactions and as a result higher sensitivities when used as sensors. However, overly thin microfibres (less than 1 µm typically) have poor mechanical strength, are less stable during measurements and additionally are very difficult to handle. It is practical to control the diameter of the microfibre to 1 µm to achieve a manageable level while retaining to the greatest extent possible the capacity of the microfibre to act as a biosensing platform.

## 3. Important Parameters for Optical Microfibre Biosensors

The performance of optical microfibre based biosensors is typically governed by a set of parameters including: robustness, strength, sensitivity, resolution, detection limit, response time, operating range, repeatability and reproducibility.

### 3.1. Robustness

Although the free-standing optical microfibre is easy to fabricate, optical microfibre based photonic components such as resonators are maintained by van der Waals or electrostatic forces at the joint area, thus they are certainly mechanically fragile, especially in a liquid environment for a bio-sensing application. In free space, the fabrication of these devices with high reliability is challenging due to problems of stability, degradation, and cleanness [21]. To enhance the robustness of an optical microfibre loop for operation in a liquid environment, an additional mechanical support is essential in order to improve its mechanical stability. As an example, an UV curable epoxy packaging technique can be adopted to embed the tapered microfibre into the UV epoxy coating which is an elegant way to solve these issues; yet, the determination of the coating thickness is a challenging issue because a thick coating layer will limit the extent of the evanescent field available for sensing, while a thin layer does not provide an appropriate level of protection for the device. Consequently, the development of 3D microcoil resonators in Teflon [22] and in low index liquid [23], a refractometric sensor based on a coated optical nanowire microcoil resonator, have been investigated and demonstrated previously [24]. Overall, the UV curable epoxy packaging technique has proved to be an important approach to improving the robustness of tapered microfibres.

### 3.2. Strength

It is well known that silica has an extremely flexible local atomic structure which is the standard deviation of the angles existing between neighboring atoms, such as Si-O-Si and O-Si-O. On the other hand, Si-O bonds have a strength (circa 621.7 kJ/mol) [25] which is comparable to the value observed for C-C bonds. These unique properties therefore result in superior macroscopic properties including a high glass-transition (circa 1410 K) and softening temperatures (1940 K), large Young modulus (73.1 GPa) and hardness value (6.5 in the Mohs scale). Moreover, because of its glassy nature, silica nanowires can potentially be manufactured in extremely long lengths: virtually infinitely long tapers can be fabricated from optical fibres. In [26], the measured ultimate strength of silica nanowire was in excess of 10 GPa and is increased for decreasing nanowire diameters. Such high ultimate strength of silica nanowires manufactured by the top-down modified flame brushing technique together with the possibility to pull silica glass based optical fibres into extremely long wires can open a wealth of applications in the macroscopic world.

### 3.3. Sensitivity

Sensitivity generally describes the change in the detected parameter as a result of a change in the measurand. For example, in the case of refractometric sensors, a change in the refractive index (measured in refractive index units—RIU) is very often understood in terms of wavelength shift relative to an initial wavelength. Therefore, this detection parameter is defined in nm, yielding the value of sensitivity of refractive index sensing in nm/RIU. However if the change is measured in the intensity domain, the detection parameter is defined in dB, therefore the value of sensitivity of refractive index sensing should be in dB/RIU.

### 3.4. Resolution of Detection System

Resolution indicates the minimal detectable change in the parameter used for detection. The resolution is related to the precision with which the measurement is made and hence is usually affected by the specifications of the detection system.

### 3.5. Detection Limit (DL) of Measurand

Detection limit (DL) represents the minimum quantity change in the parameter to monitor that can be detected by the sensor. It is related to the resolution and sensitivity by the following relationship:

DL = Resolution/Sensitivity(8)

Generally, small DLs are attractive, especially when dealing with chemical or biological compounds.

### 3.6. Response Time

Response time (τ) is typically defined as the time required for the output signal to rise from 10% to 90% of the final value, measured from the onset of the step input change in the parameter to monitor. Most of optical microfibre sensors have a very fast response time (of the order of ns) due to their compact physical size.

### 3.7. Operating Range

Operating range (OR) is the range of values of the parameter under examination, which can be measured by the biosensor, and hence by definition large OR values are highly desirable.

### 3.8. Repeatability

Repeatability is an indication of the agreement between the measured performances of the same sample taken under the same experimental conditions at different times. Normally, according to the results obtained from the experiments, two important parameters such as the standard deviation (SD) and coefficient of variation (CV %) have been determined to represent the repeatability, respectively. Good repeatability results from the stability and life-time of the biosensor head; this is more important to biosensors that can have a deviation signal because of the biological detection element.

### 3.9. Reproducibility

Reproducibility is based on the agreement between the measured performances between different bio-samples taken under the same experimental conditions. A good reproducibility is ideal for the manufacturing of multiple biosensor heads with the same specifications. To evaluate the reproducibility of optical tapered microfibre based biosensors, the quality of the tapered optical microfibre is expressed in terms of circularity and diameter of waist, as well as loss spectrum, profile of transition region *etc*. As an example, the traditional heating source for the fabrication of tapered optical microfibre is flame touch; however, the reproducibility is poor because a torch flame cannot provide a repeatable heating area size and temperature. Therefore, a ceramic microheater has been adopted as a heating source to overcome the shortcomings. The standard deviation of diameters of the tapered microfibres can be controlled down to <10% by using this fabrication method, which shows a reasonable good reproducibility of tapered optical fibre based biosensors.

## 4. Fabrication of Optical Microfibres

The microfibres introduced in this review are fabricated by tapering standard SMFs. Traditionally, if the material used to manufacture optical microfibre is in fibre form, then the microfibres can be fabricated by (1) tapering the fibre by pulling it around a sapphire rod heated by a flame torch [27]; (2) the flame-brushing technique [28]; (3) the modified flame-brushing technique [29]. The flame-brushing technique has been previously used for the fabrication of fibre tapers and couplers [28]. A small flame moves under an optical fibre that is being stretched: because of mass conservation, the heated area experiences a diameter decrease. Controlling the flame movement and the fibre stretching rate lets the taper shape be defined to an extremely high-degree of accuracy. This technique provides access to the microfibre from both pigtailed ends. Moreover, it delivers optical microfibres and nanowires with radii as small as 30 nm [21], the most uniform optical microfibres and nanowires and the lowest measured loss to date. The third fabrication method is a modified version of the flame-brushing technique [29] in which the flame torch is replaced by a different heat source to work with different types of glasses. So far, two types of heat sources have been used: a sapphire capillary tube heated by a CO_2_ laser beam [30], and a ceramic microheater [31]. This method provides optical microfibre/nanowire from a range of soft glasses with a low melting point, including lead silicates [32], bismuth silicate [32], tellurite glass [9] and chalcogenides glass [12]. The manufacturing of optical microfibre using a ceramic microheater will be introduced as follows.

A standard fibre tapering process can be described in a general as follows: (1) the coating of a short section (3–4 cm) of optical singlemode fibre is stripped off and the stripped fibre is cleaned with chemical solvents; (2) the fibre is positioned straight with its two ends (with cladding on) fixed onto two translation stages; (3) the bare fibre section is placed into/over a high-temperature heating source—the temperature adopted for heating should be above the transition temperature of silica glass (usually from 1000 to 1200 °C) to sufficiently soften the silica fibre into a viscoelastic state; (4) the two motor controlled translation stages slowly move outwards in the opposite directions to stretch the silica fibre and form it into a biconically tapered shape with a required diameter.

**Figure 4:**
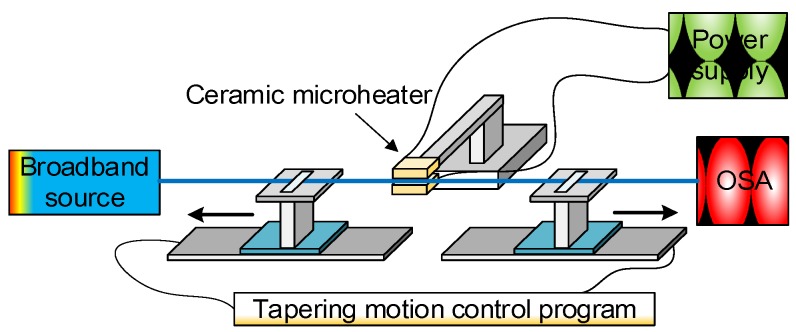
Schematic diagram of the microfibre fabrication setup based on the microheater brushing technique.

Figure 4 shows the experimental fibre tapering setup using a “modified flame brushing” technique developed at the Photonics Research Centre of Dublin Institute of Technology, Ireland for fabricating optical microfibres.

**Figure 5:**
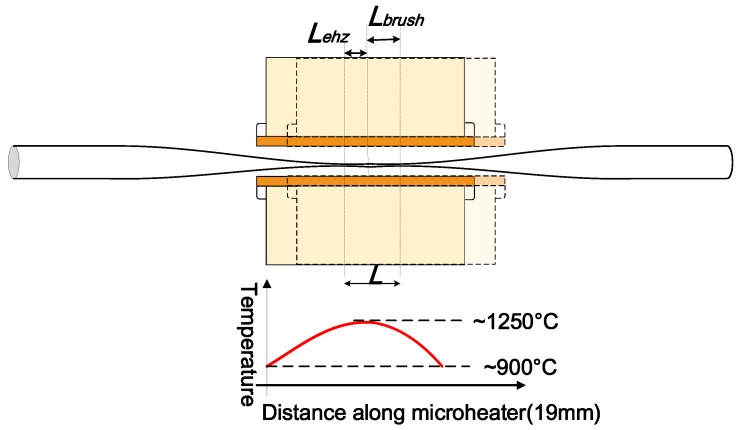
Schematic diagram of a microheater in brushing motion to illustrate the effective heating zone *L _ehz_*, microheater brushing length *L_brush_*, and the total effective heating length *L*. Temperature distribution along the heating slot of the microheater is also shown.

The setup consists of two major parts:

- **Heating source.** A ceramic microheater (CMH-7019, NTT-AT, Japan) is used to heat the optical fibre to approximately 1100~1200 °C. Silica fibres can reach a viscoelastic state around this temperature range which is suitable for tapering. The microheater is mounted on a 3-dimensional translation stage to precisely control the position of the microheater relative to the fibre. One factor that must be accounted for is that the temperature along the heat zone of the microheater is not uniform. The highest temperature occurs at the centre and gradually drops towards the edges. For instance, when the measured temperature in the centre of the microheater reaches 1250 °C, the temperature at the edges only reaches 900 °C. The temperature distribution along the heating slot of the microheater is shown in Figure 5. This quasi-Gaussian distribution of the temperature means that only a 1–2 mm heating zone at the centre of the 19 mm wide heating zone is effective for fibre tapering. This 1–2 mm long heating zone is defined as the microheater effective heat zone *L_ehz_* [7]. The length of the microfibre waist region is assumed to be equal to the length of this effective heat zone, where a simple single stretching pull of the fibre is employed. In order to fabricate microfibres with longer waist regions, the effective heating length can be extended by introducing a repeated brushing motion of the microheater into the tapering process [33]. The travel range of this repeated brushing motion is defined as microheater brushing length *L_brush_*. Thus, tapered microfibres with various waist lengths can be fabricated by varying the total effective heating length *L* = *L _ehz_* + *L_brush_* by controlling the microheater brushing length *L_brush_*. The *L _ehz_* and *L_brush_* parameters are illustrated in Figure 5.

- **Pulling rigs**. Two precision controlled linear motorized translation motion stages are used to stretch the heated fibre. A tapering motion control program, written in C++, was developed to precisely control the tapering process. By setting parameters in the program such as original fibre diameter, required microfibre waist diameter, microheater brushing length (explained above), *etc.*, microfibres with a controllable profile for both the waist and the transition regions can be fabricated. In the actual tapering process, the two motorized translation stages not only perform the pulling motion but also the microheater brushing motion. It is possible to move the microheater to perform the brushing technique but this is more difficult in practice because of positioning instability and the potential safety hazards of actually moving the microheater itself. Instead, the pulling and brushing motions are integrated in the following way. During tapering, the two motorized translation stages work together in a synchronous fashion to create an equivalent effect of a moving “brushing” microheater. At the same time, a small difference in translation speed (0.1 mm/s) is maintained between the two stages to elongate the fibre. However, the term “microheater brushing length” is still used for consistency in the following paragraphs.

The program for controlling the motions of the fabrication rig is based on the theory developed by Birks *et al.* [7], which assumes the volume of the fibre to be constant during the tapering process. The length of the uniform waist region always equals to the length of the softened fibre, which also equals to the total effective heating length *L* which was defined earlier in Figure 5. The following equation holds within these conditions [7]:

(9) $$\frac{dr_{w}}{dx}=-\frac{r_{w}}{2L}$$

where *r_w_* is the radius of the microfibre waist , and *x* is the total elongation of the fibre after tapering. During fabrication, *L* can be kept constant or varied during the tapering process. A constant *L* value is used to fabricate tapers which have relatively shallow transition regions with a decaying-exponential profile. Given an initial radius of the original fibre *r_0_*, the final waist radius of the microfibre *r_w_* is expressed as [7]:

(10) $$r_{w}\left(x\right)=r_{0}e^{\frac{-x}{2L}}$$

The profile of the transition region can be expressed as follows:

(11) $$r\left(z\right)=r_{0}e^{\frac{-z}{L}}$$

Microfibres with other types of transition region profiles can be fabricated by changing the effective heating length relative to the rate of taper elongation. This can be achieved by varying the heating length *L* with the taper elongation *x* in a real-time fashion during the tapering process. Details on calculating the waist radius and plotting the transition region profile for varying *L* can be found in Reference [7]. Tapers with such an exponentially decaying-transition profile can be considered as adiabatic. In principle, tapers are adiabatic if the transition profile meets the adiabaticity criteria described in [34]. Such optimal adiabatic tapers should be fabricated by varying the heating length *L* during the tapering process according to the relationship between *L* and *x* (taper elongation) demonstrated in [7]. However, it was experimentally proved [35] that long tapers with a decaying-exponential profile are adiabatic with loss of less than 1%.

It should be noted that discrepancies between the calculated and the actual diameters of the microfibres can occur in practice during fabrication. The temperature along the microheater slot where the fibre is inserted has a quasi-Gaussian profile as shown previously in Figure 5. This temperature profile makes it difficult to decide the exact length of the effective heat zone *L_ehz_* of the microheater which in turn affects the total effective heating length *L*. Also, the airflow in proximity of the microheater can significantly influence the temperature profile of the microheater. A small variation in *L* can lead to large errors in predicting *r_w_* since *r_w_* exponentially depends on *L*. Thus, the determination of an appropriate value for *L_ehz_* is critical for accurate prediction of the diameter of the fabricated microfibres. To do so, the actual diameters for a range of fabricated microfibres with different calculated diameters were first measured using a microscope for our case in the lab. Then different values of *L_ehz_* were tested until the discrepancy between the calculated and the experimentally measured diameters was minimized down to less than 10% for the fabricated samples.

## 5. Optical Microfibre Based Biosensors

Optical microfibres have a wide range of applications due to their ability to generate a large evanescent field which is readily accessible, the ability to tightly confine modes to the microfibre surface, and the capability to form useful configurations with small losses to allow for the development of physically compact devices. Tong *et al.* [36] provided a comprehensive review of the recent developed microfibre based photonic applications in various fields.

This section focuses on approaches utilizing the near-field interactions between the evanescent field of microfibre and the local environment as a means to underpin label-free biosensors. In this section, several typical microfibre related photonic components reported in the literatures to date are reviewed, with a mention where appropriate of the biosensing application involved. The structures covered in this review include:

(1) Straight microfibre biosensor;
(2) Microfibre tip biosensor;
(3) Evanescently coupled microfibre biosensor;
(4) Heterogeneous resonant biosensors.

### 5.1. Straight Microfibre Biosensor

A traditional straight microfibre consists of three regions (see Figure 1a): a convergent transition region with a decreasing diameter, a region of uniform waist, and a divergent transition region with an increasing diameter. The waist region is used as the sensing region because the evanescent field is at its maximum in the region of the smallest diameter and diameter is constant, meaning that the effects of changes in the surrounding environment are not position dependent. Changes in the absorption, scattering and also resonance can occur in the tapered fibre waist. Light resulting from the evanescent field of the uniform waist is collected along with the transmitted and scattered light in the divergent transition region. Changes of sensor response arising from the evanescent field are very versatile because they can be detected alone, amplified, or detected in conjunction with various optical methods. Normally, because the penetration depth is small in relation to the target analyte, the use of additional transduction mechanisms is imperative for amplification of response signals. The mechanisms used in evanescent field sensing depend on the bio-analytes and the application of the biosensor. The use of microfibre evanescent label-free biosensing has been previously investigated, including:

- **Evanescent wave absorption biosensors**
  A change in the surrounding refractive index can strongly influence the evanescent wave absorption loss of an optical microfibre. This points to a possible approach to implement label-free biosensor using such an optical microfibre. For example, if a specific antigen is to be sensed or detected, this will be possible if the presence of this antigen in the environment surrounding the optical microfibre alters the refractive index surrounding the evanescent wave of optical microfibre. It is known that antibody-antigen binding can alter local refractive index [37] and furthermore because binding can take place only for certain antibody–antigen pairing, it is possible to introduce selectivity into the detection of an antigen, by the appropriate selection of an antibody as a so called “receptor” for the antigen. Thus, to create a label-free immunosensor, one approach is to arrange for specific antibodies to adhere to the surface of optical microfibre due to physical adsorption on the fibre surface, sometimes referred to as “immobilization” of the antibody. The presence of the antibodies allows for the binding of the corresponding antigen and when the specific binding between the antibody and the antigen occurs, the surface refractive index of the optical microfibre is increased and thus detectable absorption changes for the transmission spectra occur. In 2007, Leung *et al.* [38] reported a biosensor based on a 12 μm diameter tapered optical microfibre for the detection of protein–protein interactions. The sensor could detect the binding of bovine serum albumin (BSA) to antibody that were immobilized on the tapered fibre surface with a detection limit of 100 fg/mL. To further explore the application of the optical microfibre sensor, the same research group also developed a label-free sensor for the detection of DNA [39]. In that work, a microfibre with a diameter of 5–10 μm was coated with a layer of 500 nm gold film to facilitate the immobilization of the receptor 15-mer ssDNA. The complementary 10-mer ssDNA was detected with a detection limit of 750 fM. After Leung’s work, several other label-free biosensors were developed for the diagnostics of various diseases. An example was an anti-gliadin antibody (AGAs) sensor for the diagnostic of celiac disease developed by Corres *et al.* in 2008 [40]. The sensor was fabricated by immobilizing gliadin antigen on a 20 μm diameter microfibre and could detect AGAs with a minimum detection limit of 1 ppm. Another label-free biosensor for the detection of immunoglobulin G (IgG) was developed by Tian *et al.* [41] using a nonadiabatic tapered fibre with a waist diameter of 10 μm. The microfibre was fixed in a microfluidic chip for enhanced ruggedness and a better sensor performance. The developed IgG sensor worked in a wavelength domain interrogation by detecting the dip wavelength shifts of the transmission spectra caused by the specific binding between IgG and anti-IgG. Other two nonadiabatic optical microfibre based biosensors which utilized a wavelength domain interrogation were developed by Zibaii *et al.* [42,43]. The authors first developed a biosensor for the detection of D-glucose in deionized water and for the measurement of the RI of amino acids (AAs) in carbohydrate solution [42]. The authors then studied DNA–DNA interactions to detect the hybridization of 25-mer DNA with an immobilized counterpart on the surface of a tapered microfibre [43]. In addition to the detection of DNA-DNA interactions, Bagheri *et al.* later verified that a nonadiabatic tapered microfibre also had the capability to detect intramolecular structural changes for the DNAzyme formation of particular guanine-rich oligonucleotide [44]. Apart from protein or DNA detection, a biosensor for measuring bacterial growth was developed by Zibaii *et al.* in 2010 [45]. In that paper, the surface of a tapered microfibre with a waist diameter of 6–7 μm was immobilized with Escherichia coli (*E.coli* K-12) bacteria. As the bacterial population grew, the RI on the microfibre surface increased which resulted in changes in the fibre transmission. The sensitivity for the sensor to detect bacterial growth was measured to be 60 *E.coli*·mm^−2^. Overall, changes in the absorption of the evanescent field can be detected by measuring the changes in output power of the functionalized uniform microfibre. The parameters which influence the performance of evanescent wave absorption biosensors include: diameter of microfibre, type of functionalized materials immobilized on the microfibre and length of the microfibre uniform waist region.
- **Surface Plasmon Resonance (SPR) Based Biosensors**
  Since the first demonstration of a sensing application for gas and biomolecule detection using surface plasmon resonance (SPR) in 1983 by Leidberg *et al.* [46], SPR has been a powerful tool for developing label-free biosensors. SPR consists of electron charge density oscillations propagating at the interface between a dielectric and a metal stimulated by incident light. Optical fibre based SPR sensors are simpler in construction compared with prism-based SPR sensors. Using optical microfibres can provide an even simpler solution to get access to the evanescent field. By coating a layer of metallic film on the surface of an optical microfibre, the evanescent field guided along the microfibre can be coupled to the SPR to facilitate high-performance biosensing applications. The coupled SPR shows a strong intensity and a short decay distance (200–400 nm) and the resonance frequency depends on the metal material, the microfibre parameters and the dielectric properties of the surrounding medium [47]. The first tapered optical fibre based SPR sensor was demonstrated in 1995 by Tubb *et al.* [48]. The authors coated a layer of silver film (with a thickness of 50 nm) on the surface of a tapered fibre which had a waist diameter of 10 μm. The RI sensitivity and the potential for the structure to work as a biosensor were demonstrated in that paper. After Tubb’s work, Diez *et al.* investigated a tapered fibre SPR structure fabricated by evaporating a gold film onto the surface of a wide taper which had a diameter of 30 μm to excite hybrid SPR modes with multipeaks [49,50,51,52]. Esteban *et al.* improved the performance of a similar tapered fibre SPR sensor by designing the sensing system in a reflective configuration [53]. Generally, a couple of factors dominate the performance of a microfibre SPR biosensor include thickness of metal film (in the order of the wavelength used), length of uniform microfibre and microfibre diameter; therefore, particular attention needs to be paid to these factors during the fabrication of microfibre SPR devices.
- **Localized Surface Plasmon Resonance (LSPR)**
  In recent years, instead of a thin metal film on the fibre surface, gold or silver nanoparticles were deposited on the surface of the tapered microfibre to generate localized surface plasmon resonances (LSPR) for sensing applications. Compared with SPR, LSPR has an even shorter decay distance of typically 5–30 nm [47]. This means that LSPR based sensors are less susceptible to bulk effects, thus more suitable for developing biosensors. Biomolecules trapped on the nanoparticles surface can interact with LSPRs and in turn efficiently modify the light transmission and the detected signal. The fabrication of a LSPR sensor is also simpler and more cost effective than the fabrication of an SPR sensor since nanoparticles can be deposited onto the fibre surface through physical adsorption by simply immersing a functionalized microfibre in a nanoparticle colloidal solution. The first work on immobilizing nanoparticles onto the surface of tapered optical microfibres to generate LSPR was carried out by Cui *et al.* in 2011 [54]. The effect of the size of the gold nanoparticles on the performance of the LSPR based tapered optical microfibre sensor was investigated in that paper. It was found that the sensor with larger size nanoparticles exhibited higher sensitivities in detecting RI changes in the local environment. The next year, Lin *et al.* developed a LSPR based refractive and biochemical sensor by incorporating gold nanoparticles on the surface of a 48 µm diameter tapered fibre [55]. The resonance wavelength and the transmission intensity were shown to vary linearly with the local RI changes. In 2014, Li *et al.* proposed a sensor for cancer diagnostics by immobilizing gold nanoparticles onto the surface of a 1 µm diameter tapered microfibre [56]. A detection limit of 0.2 ng/mL was achieved in the detection of alpha-fetoprotein in a phosphate-buffered saline (PBS) solution. Apart from spherical nanoparticles, nanoparticles of other shapes were also studied in conjunction with microfibres. For example, Zhang *et al.* compared the effect of two chemical solvents used for immobilizing star-shaped gold nanoparticles onto the surface of tapered optical fibres [57].
- **Structural Modification (Microfibre Grating) on Microfibre Based Biosensors**
  Structural modification on optical microfibre is of great promise due to their compactness, large evanescent field effect, low stiffness, and good flexibility. A lot of studies related to the fabrication and the potential applications of structural modification on optical microfibre in sensing and communication have been conducted in the past. Nowadays, a variety of microfibre Bragg gratings have been successfully fabricated by introducing periodic variations of either the refractive index or fibre geometries, with a wide range of techniques. By fabricating fibre Bragg gratings (FBG) within optical microfibres, the advantages of microfibres and FBGs can be combined to develop sensors with improved compactness and faster responses. Similar to conventional FBGs, microfibre FBGs can be fabricated by periodically modifying the refractive index along the microfibres. Typical techniques include inscription of photosensitive microfibres using ultraviolet (UV) lasers [58] or inscription of microfibres that are not photosensitive using femtosecond lasers [59]. FBGs can also be fabricated by direct precision milling on the surface of microfibres using a focused ion beams technique [60]. Several microfibre FBG structures have been developed for use as refractive index sensors [61,62] with diameters ranging from 2 to 6 μm. In addition to refractive index sensors, a microfibre FBG temperature sensor [62] and a force sensor [63] have also been developed. Apart from FBGs that are written in microfibres, several sensors were also developed by concatenating an FBG written in a conventional fibre with a fibre taper to create a microfibre-FBG composite structure. An accelerometer [64] and a strain sensor [65] have been developed based on such configurations. In addition to FBGs, long period gratings (LPG) can also be written in microfibres. Xuan *et al.* demonstrated a microfibre based LPG fabricated using a focused high frequency CO_2_ laser [66]. Both the temperature and RI sensitivity of that microfibre LPG were investigated. Very recently, *in-situ* DNA hybridization detection using a reflective microfibre grating based label-free biosensor and poly-L-lysine (PLL) monolayer-modified microfibre Bragg grating label-free biosensor for a specific DNA detection were demonstrated by D. Sun *et al.* [67,68]. The microfibre grating label-free biosensor presented in [67] can detect the presence of the DNA hybridization with high specificity, in various concentrations of target DNA solutions, with a lowest detectable concentration of 0.5 µM. In summary, the structural modification (such as microfibre grating) based label-free biosensors is a good candidate for rapid and highly sensitive detection in microliter volumes of analytes at low concentrations (sub-microliter dose) in medicine, chemical and environmental monitoring.

### 5.2. Microfibre Tip Biosensor

**Figure 6:**
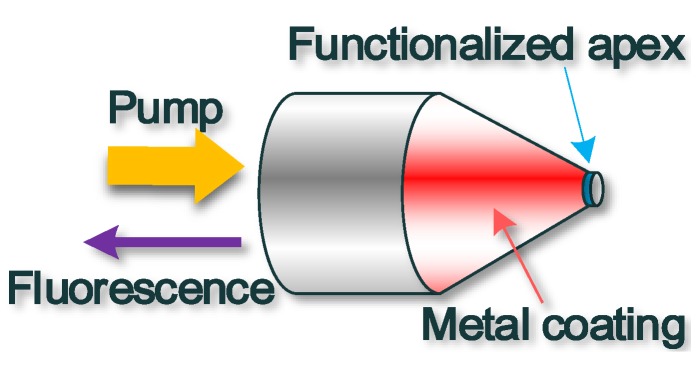
Schematic of an optical microfibre tip sensor, light injected at the fibre pigtail pumps the functionalised coating, which emits fluorescent light that is collected when it is in proximity of the target compound.

Previously, microfibre tips were used for trapping micro-particles, for micro-manipulation or for coupling light in/out optical photonic integrated circuits. Recently, optical microfibres have exploited the extremely small end-face cross section (sub-200 nm) to analyse minute sensing areas, such as cell components. The advantage of using a microfibre tip as a biosensor head is the possibility of a spatial operation, especially in confined areas. In order to achieve extreme confinement, optical microfibre tip sensors were manufactured from very thin metal film coated optical microfibre tips. The end face was left uncoated (see Figure 6 below) and it underwent functionalization to facilitate covalent bonding of the targeted bio-molecule. The functionalization compound is excited using the longitudinal evanescent field generated inside the coated tip, thus only molecules in the immediate proximity of the optical microfibre apex aperture are affected by the optical field. Functionalization is generally carried out in three steps: silanization, activation and bonding of a biorecognition agent (usually antigens or antibodies with a fluorophore). The first optical microfibre employed for intracellular sensing was used to measure pH [69]. Functionalization was carried out using a chromophore (N-fluoresceinyl-acrylamide) in an acrylic copolymer. The chromophore was excited using blue light laser light and fluorescence was collected at 490, 540 and 610 nm. pH values in the range 4 to 9 were measured from the ratios between fluorescence peaks at different wavelengths (540/490 nm and 540/610 nm). The sensor response time was of the order of τ~100 ms. Biochemical compounds measured *in vivo*, in live cells include benzopyrene tetrol and benzo[α]pyrene [70], Caspase-9 [71], cytochrome c [72], a telomerase [73] and DNA sequences [74]. The small sensor size allows for *in-situ* detection of specific chemicals without visible damage to the cell. DNA specific-actin mRNA strands were detected functionalizing the apex with molecular beacons [74]; this method has been proved capable of recognizing differences in single couple of bases. Ion concentrations were measured by functionalizing the sensor apex with plasticized polymeric membranes embedding ion complexation agents and fluorescent dyes. Potassium [72] and calcium [75] concentrations were detected using valinomycin and Fura-2/AM or fluo-3/AM calcium-dyes as highly-selective ligands. Other compounds measured in live cells include oxygen [76], nitrites [77], chloride ion [77], nitric oxide [78] and glutamate [79]. At the moment, the apex size is limited by the tip transmission efficiency. For tips smaller than 50 nm, the transmissivities less than 10^−6^ have been recorded [80]. A way to increase the transmissivity by orders of magnitude relies on the exploitation of surface plasmons: by converting light into surface plasmons and then back into light, confinements to spot sizes smaller than 10 nm should be possible [81], with extraordinary benefits for the biosensor size.

### 5.3. Evanescently Coupled Microfibre Biosensor

Evanescently coupled microfibres, normally in the form of optical microfibre coupler (OMC), were first demonstrated by Y. Jung in 2009 [82]. An OMC has a structure very similar to that of a conventional fused optical fibre coupler, which was originally developed at the end of 1970s [83,84]. The main difference between the two structures is that compared with a conventional fibre coupler, an OMC has a much smaller diameter, usually of a few micrometers. Due to the small diameters and the strong evanescent fields, OMCs are more sensitive to local environmental changes and thus have strong potential to be used to develop sensors.

**Figure 7:**
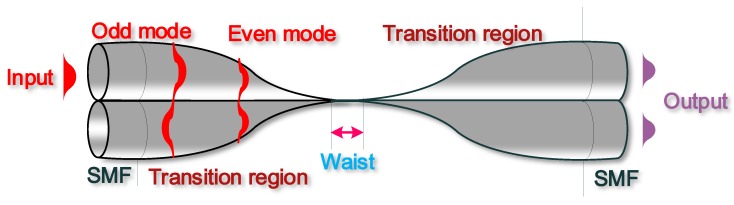
Schematic diagram of an OMC consisting of two fused microfibres with a uniform waist region and two transition regions. Light launched into one input port can be coupled and split between two output ports due to the superposition between the even and odd modes.

The structure of a typical OMC is shown in Figure 7 and consists of two identical microfibres with the entire waist region and part of the transition regions fused together:

Depending on the taper fabrication temperature [85], various degrees of fusion can be achieved between the microfibres within the OMCs [86,87]. For weakly fused OMCs, both fused microfibres roughly maintain their original cross-section geometry throughout the entire tapering process, thus such OMCs have a dumbbell-shaped cross section. In this case, the aspect ratio, defined as the ratio between the total width and the height of the cross section at the OMC waist region, is usually larger than 1.8 [85]. For strongly fused OMCs, the cross-section geometry is altered in a way that the distance between the two microfibre axes reduces faster than the decrease of the fibre diameters. The resulting coupler can have elliptical or circular shape cross sections [87]. The aspect ratios for such strongly fused OMCs can vary between 1.0 and 1.8. Compared with weakly fused couplers, strongly fused couplers require shorter coupling lengths and larger critical diameters in order to achieve a complete power transfer from one fused fibre to the other. In addition, strongly fused couplers also show weaker dependence on polarization and surrounding RI changes. Therefore, strongly fused couplers are more suited to work as beam combiners and splitters for optical fibre communications. In contrast, weakly fused couplers show stronger dependency on surrounding refractive indices than that of the strongly fused ones and are more suited to work as sensing elements.

In various microfibre based sensing applications, the detection of the analyte relies on the detection of the RI changes in the local environment. To verify the feasibility of using an OMC structure as a biosensor, the sensitivity of the OMC to detect refractive index changes in the local environment was investigated experimentally [88]. The experimental results showed that the fabricated sensor has an average sensitivity of circa 2723 nm/RIU over the entire measured refractive index range from 1.3340 to 1.3800. Furthermore, a high sensitivity of 4155 nm/RIU was obtained for the RI range from 1.3340 to 1.3515. Given that the surrounding refractive index is usually modified by biomolecules adsorbed on the fibre surface [89], the OMC has the potential to work as an ultra-high sensitive biosensor.

**Figure 8:**
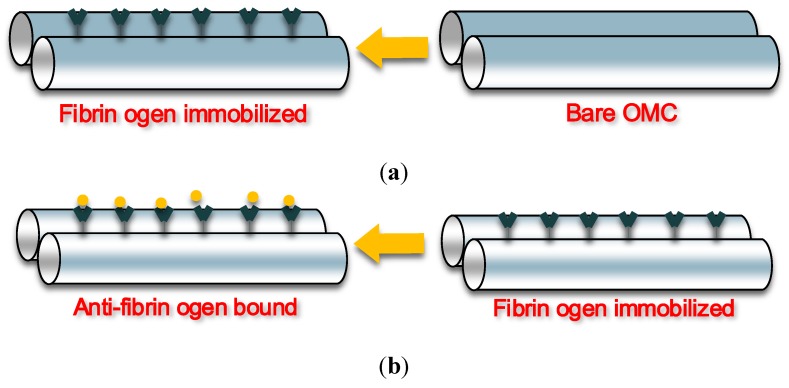
(**a**) Immobilization and (**b**) anti-fibrinogen binding with the immobilized fibrinogen.

Recently, a label-free immunosensor based on an optical microfibre coupler structure was investigated for detecting specific antibodies using a fibrinogen antigen-antibody pair [90]. To illustrate the biosensing mechanism of this sensor, Figure 8 schematically shows (a) the immobilization process and (b) the process of anti-fibrinogen binding with the immobilized fibrinogen. As shown in the figures, the sensor has been fabricated by immobilizing fibrinogen on the sensing surface of the embedded OMC. Experimental results showed that the OMC based immunosensor is capable of detecting anti-fibrinogen and that the detected signal is proportional to the amount of the anti-fibrinogen present. This OMC based biosensor offers a reliable, compact and simple solution in current immunoassay sensing applications.

Ismaeel *et al.* [91,92] demonstrated a DNA sensing application using both the microfibre coupler and the multi-port microcoil resonator structures. For the straight OMC, the OMC was first processed by the silanization process, and then the OMC was functionalized by using a 1% solution of distilled trimethoxysilylpropyldiethylenetriamine in 1 mM acetic acid in deionized (DI) water for 20 min at room temperature, followed by a surface modification with the heterobifunctional crosslinker, N-(-maleimidobutryloxy) succinimide ester (GMBS), and prepared as 1 mM solutions. Crosslinkers were dissolved in 100 µL dimethyl sulphoxide (DMSO) and diluted to 30 mL in 80:20 MeOH:DMSO. The silyl functionalized coupler was immersed in the GMBS crosslinker solution for 2 hours at room temperature. At last, the attachment of the DNA:3-end labelled thiol-DNA oligonucleotide in deaerated 10 mM HEPES and 5 mM EDTA buffer (pH = 6.6) was applied on the surface of the charged OMC to see the changes on the wavelength domain. Immobilization caused 4.7 nm wavelength shift while hybridization caused an extra 5.2 nm wavelength shift have been observed in total. The device promises a high sensitivity up to 200 nm /RIU, and a low detection resolution circa 10^−6^ RIU.

Additionally, a multi-port microcoil resonator was fabricated by coiling an OMC around a PMMA tube with a diameter of circa 1 mm which was then embedded in Teflon [92]. The PMMA rod was then carefully dissolved by acetone forming a hollow core Teflon cylinder that has the coiled microfibre in its inner surface, ready to be functionalized with biomaterial.

### 5.4. Heterogeneous Resonant Biosensors

Heterogeneous microresonators represent a large group of optical microfibre bio-applications. This group of resonant sensors exploits optical microfibres only to inject/extract light from high-Q micro-resonators (like microspheres, microtoroids, microcapillaries and microbottle resonators) which are in contact as a coupling waveguide. Evanescent sensing in these types of high-Q microresonators has been used to monitor chemical and biological elements positioned in proximity of the resonator surface. Here, the analyte alters the resonance wavelength of the microresonator and by monitoring the wavelength shift it is possible to determine the analyte concentration with a high degree of accuracy. In general, high-Q factors are associated with very high sensitivities, but also with very difficult input/output light coupling. Optical microfibres have proved the best solution for coupling light in/out microresonators, providing efficiencies in excess of 90% [16].

**Figure 9:**
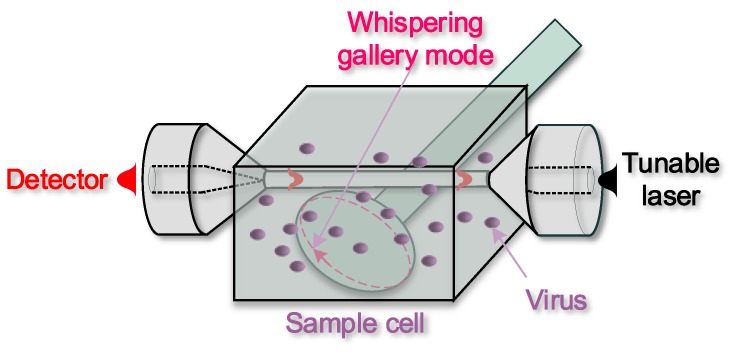
Schematic of a heterogeneous resonant sensor: an optical microfibre couples light into the resonant whispering gallery mode of a microsphere (dashed line), which is affected by the surrounding fluid.

Because of their high Q and of their extreme ease of fabrication, microspheres have been widely used for biological detection; a schematic diagram of a sensing system using microspheres is shown in Figure 9. In 2008, F. Vollmer has first shown the possibility to achieve a single virus of Influenza A and to measure its mass (~0.52 fg) by using a microsphere with radius of 39 μm and light at the wavelength λ = 763 nm [93]. Streptavidin was detected using a microsphere resonator with Q~2 × 10^6^ functionalized with bovine serum albumin biotin [94]. Specific DNA strands were detected using multiple spheres coated first with a dextran-biotin hydrogel and then with a mixture of biotinylated 27-mer oligonucleotides and streptavidin [95]. Single cylindrical bacteria were detected exploiting microsphere surface adsorption and the related resonance bandwidth broadening associated to increased scattering losses: a detection limit of 44 bacteria was achieved for Escherichia coli [96]. Toroidal microresonators have been also investigated broadly because of their extremely high-Q factors. Single molecules of interleukin-2 (a cytokine) [97] were detected functionalizing the silica surface to bind the target molecule and by measuring the resonance wavelength shift, concentrations from 5 aM (5 × 10^–18^) to 1 µM (1 × 10^–6^) were recorded. Very recently [98], a whispering gallery mode microlaser-based real-time and label-free detection method has been reported that can detect individual 15-nm-radius polystyrene nanoparticles, 10-nm gold nanoparticles and influenza A virions in air, and 30 nm polystyrene nanoparticles in water, by monitoring an ultra-narrow linewidth lasing mode splitting and interference effects. Given the unique characteristics of line narrowing, high confinement of light on resonator and luminescence enhancement offered by this heterogeneous microresonator, it can be a very successful next generation microsystem biodetection technology.

## 6. Conclusions

In the past decade, optical microfibre based photonic component/devices have attracted increasing interest because of their tremendous potential in a wide range of fields ranging from telecommunications to sensing applications. In this review, we analysed the traditional fabrication technique using a ceramic microheater and the optical and mechanical properties of microfibres. Because of their flexibility, easy connectivity and extraordinary properties, optical microfibre based photonic components have been used in a wide range of applications, which have been grouped according to the property that they exploit: transition regions, confinement or the evanescent field. Table 1 below lists some of the most prominent research work cited in this review:

**Table 1:** Comparison of performances in each tapered optical microfibre design category for biosensing.

Optical microfibre (OM) Biosensor Configuration	OM Size (Waist)	Biological Target	Detection Limit	Reference
Straight OM	12 μm	Bovine serum albumin	100 fg/mL	Leung *et al.* [38]
	5–10 μm	10-mer ssDNA	750 fM	Leung *et al.* [39]
	20 μm	Anti-gliadin antibodies	1 ppm	Corres *et al.* [40]
	10 μm	Immunoglobulin G	N/A	Tian *et al.* [41]
	6–7 μm	Bacteria E. coli K-12	10 bacteria bound	Zibaii *et al.* [42]
SPR based sensors	10 μm	N/A	RIU 5 × 10^−4^	Tubb *et al.* [48]
	30 μm	N/A	RIU 10^−5^	Díez *et al.* [52]
LSPR based sensors	48 µm	Anti-DNP	4.8 pM	Lin *et al.* [55]
	1 µm	Alpha-fetoprotein	0.2 ng/mL	Li *et al.* [56]
Microfibre grating based sensor	3–5 µm	ssDNA	0.5 µM	Sun *et al.* [67]
Microfibre tip sensors	200 nm	Benzopyrene tetrol & benzo[α]pyrene	9.6 ± 2 pM	Vo-Dinh *et al.* [70]
	150 nm	Caspase-9	N/A	Kasili *et al.* [71]
	150 nm	Cytochrome c	N/A	Song *et al.* [72]
	500 nm	A telomerase	N/A	Zheng *et al.* [73]
Evanescently coupled microfibre sensors	2 µm	Anti-fibrinogen	25 µg/mL	Bo *et al.* [90]
	5 µm	DNA:3-end labelled thiol-DNA oligonucleotide	10^−6^ RIU	Ismaeel *et al.* [91,92]
Heterogeneous resonant sensors	2 µm	Human InfA virus A/PR/8/34	5.2 × 10^−16^ g	Vollmer *et al.* [93]
	4 µm	Streptavidin	0.01 mg/mL	Vollmer *et al.* [94]
	2–4 µm	E. Coli bacteria	~44 bacteria bound	Ren *et al.* [96]

Optical microfibre based biosensors are promising alternatives to traditional immunological methods for biomolecule measurements. Unlabeled DNA and protein targets can be detected by monitoring the changes of various optical transduction mechanisms, such as refractive index, absorption, fluorescence and surface plasmon resonance, when a target molecule binds to an immobilized optical microfibre.

As the applications of optical microfibre are evolving, new efforts on enhancing the performance sensitivity and selectivity of optical microfibre based label-free biosensors will be investigated. For example, it appears that methods based on SPR technique have reached a plateau in terms of detection limit, and one possible future direction is to combine multiple methods, such as micromachining and the nano-milling technique. Therefore, the SPR signal can be enhanced significantly. On the other hand, improved surface chemical modification and stability of the recognition molecule can also increase the sensitivity and robustness of optical microfibre based label-free biosensors, especially for evanescent wave absorption biosensors because they are the most sensitive when molecules are bound with the functionalized thin film on the surface of the optical microfibre. As was shown in this review, there is a solid foundation of work to support the use of tapered optical microfibres in a wide variety of biosensing applications. Given their unique properties and promising advantages, it is likely that tapered optical microfibres will still remain a popular choice among scientific researchers and also commercially for detection of biological targets. Moreover, with options for an improved wavelength operating coverage, particularly for the mid-infrared wavelength range—afforded by new glass material types and advances in inherent molecular selectivity which allows qualitative and quantitative analysis of various chemical and biological samples, and also offers access to fundamental vibrational fingerprint absorptions of organic molecules—the future opportunities for optical microfibre based photonic component related bio-technology research and further commercialization appear very bright indeed.
